# G91-deletion in βA3/A1-crystallin induces cellular and molecular changes in mouse lenses leading to congenital cataract development

**DOI:** 10.1371/journal.pone.0326305

**Published:** 2025-07-07

**Authors:** Akosua K. Boateng, Roy Joseph, Om P. Srivastava

**Affiliations:** Department of Optometry and Vision Science, School of Optometry, University of Alabama at Birmingham, Birmingham, Alabama, United States of America; Tsinghua University School of Life Sciences, CHINA

## Abstract

CRYβA1-ΔG91 (βA3ΔG91) is a mutational hotspot in CRYβA1, which causes autosomal dominant congenital nuclear cataract in humans and mice. Previous *in-vitro* studies of recombinant βA3ΔG91 showed defective folding, decreased solubility, and aberrant oligomerization of βA3ΔG91 with other crystallins. Emerging evidence demonstrates an association between autophagy and βA3ΔG91-induced congenital cataracts. To gain further understanding of the molecular mechanism of congenital cataract development in βA3ΔG91 mice, we examined the βA3ΔG91- vs WT- lenses for complete gene profiling, lens epithelial cell (LEC) proliferation and migration, and lens epithelial-fiber cell differentiation. We also determined the changes in crystallin proteomic profiles in water-soluble, water-insoluble-urea-soluble, and water-insoluble-urea-insoluble fractions. Our results show that relative to WT lenses, the βA3ΔG91 lenses showed: (A) downregulation of genes associated with LECs proliferation and migration (B) abnormal suture line pattern, (C) significant reduction in proliferation and migration of LECs, (D) abnormal F-actin distribution, (E) increased high molecular weight (HMW) peak, and (F) insolubilization and degradation of crystallins and other lens proteins. Together, these defects contribute to the formation of the lens opacity in βA3ΔG91 mice lenses.

## Introduction

The crystalline lens has the ability to change shape for variable focusing (accommodation). This function hinges on the prerequisite of lens transparency, which is dependent on several biological parameters.

Maintaining lens transparency requires a tightly regulated series of lens biological properties: i.e., high solubility of lens crystallins [1]; the unique interactions among crystallins [2–4] with almost no protein turnover [5]; specialized metabolism [6]; cellular homeostasis among epithelial and fiber cells [7], and orderly terminal differentiation of epithelial to fiber cells with precise organelle loss [8].

Lens epithelial cells (LECs) are essential for lens growth, development, and transparency. As the most metabolically active component of the lens, they regulate key cellular processes, including proliferation, migration, and fiber cell differentiation [9]. Thus, LEC integrity needs to be preserved, as disruptions can compromise lens transparency.

Lens epithelial-cell-to-fiber-cell differentiation is initiated when the lens epithelial cells at the germinative zone proliferate and then migrate to the equatorial region, where they begin to differentiate into fiber cells. These newly formed fiber cells continually appose old fiber cells. Due to this highly regulated process, the ends of the fiber cells symmetrically align to generate suture lines [21].

The vertebrate lens contains α-, β- and γ-crystallins as the major structural proteins. In their native state, α- and β-crystallins exist as oligomers, whereas γ-crystallin is a monomer. α-crystallins consist of two subunits (CRYαA and CRYαB), which act as small heat shock proteins with chaperone-like properties to prevent the aggregation of misfolded or denatured β- and γ-crystallins. The vertebrate lens expresses seven different β-crystallins, which can self-associate or interact with each other to form oligomers. β-crystallins are made up of four acidic (CRYβA) and three basic (CRYβB) subunits [10,11].

βA3/A1-crystallin exists as βA3- and βA1-crystallins. They are derived from a single CRYβA1 gene via alternate translation [12]. Structurally, βA3/A1-crystallin has six exons which encode for four Greek key motifs that fold into two domains, connected by a short peptide [12]. The distinctive Greek key pattern of β- and γ-crystallins is a structural requirement for the maintenance of structural stability and lens transparency [13].

Apart from the lens, βA3/A1-crystallin is also expressed in the retinal pigment epithelium (RPE), retinal astrocytes, and the brain [14–17]. Interestingly, in these tissues, βA3/A1-crystallin plays a role in maintaining epithelial cell properties like proliferation and migration [18] and acts as an autophagy regulator [14,16,17,19]. Our recent studies have also shown that an absent or dysfunctional βA3/A1-crystallin in murine lenses causes autophagy disruption and congenital cataract development [20–22].

Congenital cataracts cause approximately 10% of childhood blindness worldwide [23]. These cataracts are mostly caused by autosomal dominant mutations [24]. Intrinsic mutations in lens crystallins represent a major causative factor in cataract development [25]. Several mutations in the CRYβA1 gene have been linked to autosomal dominant congenital cataracts [26–37]. Among them is the commonly occurring in-frame deletion of a highly conserved Gly-91 within the second Greek key motif (named βA3ΔG91), which causes congenital nuclear cataracts. Evidence from recombinant and *in-vivo* βA3ΔG91 studies shows defective refolding, loss of solubility, aberrant oligomerization with other crystallins [33,38], and disruption of autophagy, respectively [21]. Although G91del leads to loss of βA3/A1-crystallin structural integrity, and therefore, possibly renders it a dysfunctional crystallin *in-vivo* [26], the precise molecular mechanism of cataract development remains unknown.

In this present study, we have generated a βA3ΔG91 knock-in mouse model using CRISPR-Cas9 methodology, which showed congenital nuclear cataract and dysregulation of autophagy [21]. To gain further understanding of the molecular mechanism of congenital cataract development, the aim for this study was to determine the phenotypic characteristics and examine the changes in solubility of crystallins in βA3ΔG91 relative to wild-type (WT) mouse lenses. We utilized cellular, transcriptomic, and proteomic approaches to determine the following relative structural and molecular changes in lenses of βA3ΔG91 mice relative to wild-type mice: (A) transcriptomic profiles, (B) lens suture line patterns, (C) lens epithelial cell proliferation, migration and lens fiber cell organization, and (D) relative crystallin expression levels and their distributional changes from water-soluble to water-insoluble protein fractions. The results show that relative to wild-type lenses, βA3ΔG91 lenses showed (A) downregulation of genes associated with LECs proliferation and migration, (B) abnormal suture lines, (C) decreased cell proliferation and migration, and (D) increased insolubilization of βA3/A1-crystallin along with its potential interacting-crystallins.

## Materials and methods

### Animals

We generated the βA3ΔG91 knock-in mouse model as described previously [21]. Mice were kept in a controlled, pathogen-free environment with a 12/12 h light-dark cycle. They were given standard rodent chow and unlimited access to water while being housed in plastic cages with standard rodent bedding at the University of Alabama at Birmingham (UAB). Animal husbandry and procedures were performed within the guidelines of the UAB Research Ethics Committee and followed the Association of Research in Vision and Ophthalmology Statement for the Use of Animals in Ophthalmic and Vision Research. The experimental comparison involved one-month-old βA3ΔG91 and age-matched C57Bl/6J wild-type (WT) mice. Mice were euthanized by inhalation of CO_2_, which is the standard method recommended for use with mice by the UAB animal care facility. All precautions were taken to minimize the suffering and pain of the animals during tail snipping for genotyping.

### RNA-sequencing and ingenuity pathway analyses

RNA was extracted from 1-month old lenses of WT- and βA3ΔG91 mice using the Trizol method. RNA quality was assessed using the Agilent 2100 Bioanalyzer (Agilent, Santa Clara, CA). RNA with an RNA Integrity Number (RIN) of ≥ 7.0 was used for sequencing library preparation. The RNA was converted to a sequencing ready library using the NEBNext Ultra II Directional RNA library kit with polyA selection as per the manufacturer’s instructions (NEB, Ipswich, MA). The cDNA libraries were quantified using qPCR in a Roche LightCycler 480 with the Kapa Biosystems kit for Illumina library quantitation (Kapa Biosystems, Woburn, MA) prior to cluster generation. mRNA-sequencing was performed on the Illumina NovaSeq6000 as described by the manufacturer (Illumina Inc., San Diego, CA). Raw sequence FASTQ files were first trimmed to remove primer adapters using Trim Galore (version 0.6.10). STAR (version 2.7.10a_alpha_220601) was then used to align the trimmed RNA-Seq FASTQ reads to the Gencode mouse reference genome (GRcm39 Release M32) [39]. Following alignment, HTSeq-count (version 2.0.2) estimated the transcript abundances to each gene [40]. Normalization and differential expression were then applied to the count files using DESeq2 [41]. Pathway analysis was performed using the Ingenuity Pathway Analysis software (Qiagen). Hierarchical clusters and volcano plots were generated using ShinyGO and MATLAB respectively [42].

### Lens suture line assessment

Brightfield micrographs of lens suture lines were captured using a Leica automated inverted microscope (DMI4000B). Lenses of one-month-old βA3ΔG91 and WT mice were isolated from enucleated eyes and examined in PBS at room temperature (RT) for suture line patterns using the above-described microscope.

### Histology and Immunofluorescence

Tissue sectioning followed by immunofluorescence analyses with desired antibodies were performed as previously described by us [21]**.** Briefly, eyes from one-month-old βA3ΔG91 and WT mice were enucleated and immediately embedded in Tissue-Tek O.C.T compound and stored in −80^0^C. Lens sections (16 µm) were fixed in 4% paraformaldehyde (PFA) for 10 min and blocked in 5% goat serum and 1% BSA in PBS for 1 h before overnight incubation with primary antibody. The primary antibodies used in this study were: anti-BrdU (Millipore Sigma, B8434), and Rhodamine-conjugated anti-phalloidin (Cytoskeleton Inc., PHDR1). Secondary antibody (Invitrogen) incubation was done for 1 hour in the dark at RT, and lens sections were counterstained with DAPI-containing mounting medium. Photographs were taken with a Nikon AX-R Laser Confocal Microscope and signal intensities were quantified using Image J.

### BrdU-labeling

One-month-old WT and βA3ΔG91 mice were injected intraperitoneally with 100 µg BrdU per gram of mouse body weight. Mice were sacrificed after 24 h and the eyes were enucleated and immediately placed in OCT media and kept on dry ice for 1–2 h and then transferred to −80^0^C. Lens sections of 16 µm thickness were stained with BrdU antibody overnight following the immunofluorescence protocol as stated above. Prior to blocking, the DNA hydrolysis step was performed with 1.5 M HCl for 40 min and then neutralized with 0.2 M sodium borate buffer for 15 min. BrdU-labeled immunofluorescent cells were viewed using Nikon AX-R Laser Confocal microscope and signal intensities were quantified using Image J.

### Scratch and Transwell migration assays

Cell migration was evaluated by the scratch/wound healing and transwell assays. To perform the wound healing assay, primary LECs from 1-month-old lenses were first grown to confluency in DMEM containing 1% FBS in 6-well plates. Next, the confluent layers of LECs from three groups each of both WT and βA3ΔG91 were scraped using a sterile 200µl pipette tip. At 0-, 24-, 48-, 72-, and 96- h, images of scratch wounds were captured using a Leica automated inverted microscope (DMI4000B), and the changes in the wound width were measured using ImageJ. To account for potential variations in the initial scratch width at 0h, the scratch width at each time point was normalized to the width at 0h. Relative wound closure rate was calculated as


$$=\left(\frac{woundwidthatspecifictimepoint}{{woundwidthattimepoint}_{0}}\right).$$

The transwell migration assay was performed using the Corning transwell chambers (8 µm pore size, Corning Inc.). LECs (10^5^ cells) from WT and βA3ΔG91 mice were seeded into the upper chamber of the transwell insert in 500 µl serum-free media and the lower chambers were filled with 1 ml of media containing 20% FBS+DMEM. The chamber was incubated at 37°C for 24 and 72 h. After the incubations, the migrated cells were fixed and stained with Hoechst 33342. Images of 10 random areas were taken with a fluorescent microscope, and cells were counted using ImageJ.

### Molecular modeling

The native βA3-crystallin and βA3ΔG91 amino acid sequences were submitted in FASTA format to the Zhang lab I-TASSER (Iterative Threading ASSEmbly Refinement) on-line server to predict protein structures [43]. Briefly the I-TASSER software uses structural templates from the protein data bank (PDB) and assembles the structural fragments from these templates into full-length models.

### Prediction of amyloidogenic regions

The FoldAmyloid software was utilized to predict amyloidogenic regions within the amino acid sequences of the native βA3-crystallin and βA3ΔG91. In this process, the amino acid sequences of βA3-crystallin and βA3ΔG91 were uploaded into the software in FASTA format, and the prediction process was initiated through the software’s interface. FoldAmyloid evaluates the aggregation potential of individual amino acids by calculating probabilities of backbone-backbone hydrogen bond formation and analyzing other key physicochemical properties [44]. This analysis allows FoldAmyloid to effectively classify amyloidogenic peptides within the sequences of interest.

### Thioflavin staining

Thioflavin S staining was performed in the tissue sections of 1-month-old mice lenses from WT and βA3ΔG91, respectively. The staining was performed as per the manufacturer’s protocol (Vitrovivo Biotech LLC, Rockville, MD) (VB-3035). After the thioflavin staining, the sections were incubated with nuclear stain for 5 minutes, followed by washing in PBS. The sections were mounted using mounting media and covered using a coverslip. This was followed by an analysis of amyloid staining (green fluorescence) using a Nikon AX-R Laser Confocal Microscope (VSRC, UAB) [45].

### High-Performance Liquid Chromatography (HPLC)

Two lenses were dissected from 1-month-old βA3ΔG91 and age-matched WT mice and homogenized in buffer A (5mM Tris-HCl, 1 mM EDTA, 1mM DTT, pH 7.8) containing protease inhibitor cocktail (Roche). The lens homogenates were then centrifuged at 14,000 x g for 15 min to separate the water-soluble (WS) and water-insoluble (WI) fractions. The supernatant (WS-fraction) was collected, and the pellet was resuspended in the same buffer and centrifuged as described above. The supernatants were then pooled together. The water-soluble fraction was analyzed by HPLC using TSKgel G4000PWxl column (Tosoh biosciences) coupled to an online UV detector.

### Protein extraction for mass spectrometric analyses

Lens proteins were extracted from four lenses each from 1-month-old WT and βA3ΔG91 mice and separated into water-soluble (WS), water-insoluble-urea-soluble (WI-US), and water-insoluble-urea-insoluble (WI-UI) fractions, as described previously by us [22,46]. During the fractionation of proteins, equal volumes of each buffer were used, and protein concentrations in the three fractions of the two types of lenses were determined using the BCA assay kit (Pierce Biotechnology, Rockford, IL). Equal volumes of lens proteins from WS-, WI-US- and WI-UI- fractions were electrophoretically separated by SDS-PAGE using 12% polyacrylamide gels and stained with the Coomassie blue dye.

### Mass Spectrometric Analyses

(A) In-gel Digestion:

Desired SDS-PAGE gel bands were excised, and excess stain was removed by an overnight wash with 50% 100 mM ammonium bicarbonate/50% acetonitrile. After destaining, disulfide bonds were reduced by 25 mM dithiothreitol at 50^o^C for 30 min. Alkylation of the free thiol groups was carried out with 55 mM iodoacetamide for 30 min in the dark. The excess alkylating agent was removed, and the gel pieces were washed twice with 100 mM ammonium bicarbonate for 30 min. The gel pieces were evaporated to dryness in a SpeedVac (Savant) before the addition of trypsin. A 12.5 ng/µl concentration of trypsin (Promega Gold Mass Spectrometry Grade) was added to each gel sample and incubated overnight at 37^o^C. Peptides were extracted from the gel pieces using a 1:1 mixture of 1% formic acid and acetonitrile twice for 15 min and extracts were pooled and evaporated to dryness. The samples were then re-suspended in 60 µl of 0.1% formic acid prior to mass spectrometric analysis.

(B) Ultra Performance Liquid Chromatography-MS/MS (UPLC-MS/MS) Analysis:

During analyses, an aliquot (30 µL) of each sample was loaded onto a Phenomenex 2.1 x 100 mm, 1.6 μm Luna Omega, 80 Å reverse-phase column (Torrance, CA). The mobile phases were: (A) ddH_2_O with 0.1% formic acid and (B) acetonitrile with 0.1% formic acid. A linear gradient of 5–50% mobile phase B for 10 minutes, then 50–98% B until 11 minutes with a 1-minute hold, then re-equilibration at initial conditions for 8 minutes was done using an Exion UHPLC (Sciex, Toronto, Ontario), and at a flow rate of 400µl/min. The mobile phases were: (A) ddH_2_O with 0.1% formic acid and (B) acetonitrile with 0.1% formic acid, respectively. The SCIEX 5600 Triple-Tof mass spectrometer (SCIEX, Toronto, Canada) was used to analyze the peptide profiles. The IonSpray voltages for positive modes was + 5500 V and the declustering potential was 80 V. GS1/GS2 and curtain gases were set at 40 psi and 25 psi, respectively. The interface heater temperature was 400^o^C. Eluted peptides were subjected to a time-of-flight survey scan from 400–1250 m/z to determine the top ten most intense ions for MS/MS analysis. Product ion time-of-flight scans at 50 msec were carried out to obtain the tandem mass spectra of the selected parent ions over the range from m/z 50–1500. Spectra are centroided and de-isotoped by Analyst software, version TF 1.81 (Sciex, Toronto, Ontario). An α-galactosidase trypsin digest was used to establish and confirm the mass accuracy of the mass spectrometer.

(C) Protein Pilot 4.5 Search Queries:

The tandem mass spectrometry data were processed to provide protein identifications using an in-house Protein Pilot 4.5 search engine (SCIEX) using the Mus musculus UniProt protein database and using a trypsin digestion parameter.

(D) Spectral Count Quantification

Following Mass Spectrometry analyses, with a confidence score set to at least 95%, spectral counts were computed by summing the number of MS/MS identifications. The spectrum count normalization procedure in Scaffold was used (Scaffold Proteome Software Inc, Portland, OR). Briefly, the normalized spectral count for βA3/A1-crystallin was calculated as:


$$spectrumcount*\frac{averagespectralcountbetweenWTand\beta A3\Delta G91}{totalspectralcountpergroup}$$

Normalized spectral counts of all proteins from mass spectrometry analysis are listed in Tables 1 and 2.

**Table 1:** Mass spectrometric analyses of crystallins of Mr 20–37 kDa.

1A: Normalized spectral counts for WS Crystallins			
	WT	βA3ΔG91	Difference (βA3ΔG91-WT)
γS-crystallin	2.16	2.79	0.63
βA1-crystallin	17.29	7.45	−9.84
γB-crystallin	4.32	4.65	0.33
αB-crystallin	2.16	3.72	1.56
αA-crystallin	23.77	19.54	−4.23
βB2-crystallin	6.48	8.38	1.89
γ F,E,D-crystallins	5.40	2.79	−2.61
βB3-crystallin	12.97	18.61	5.65
βA4-crystallin	1.08	1.86	0.78
βB1-crystallin	73.47	57.70	−15.77
βA2-crystallin	0.00	0.93	0.93
**1B: Normalized spectral counts for WI-US Crystallins**			
γS-crystallin	0.00	1.24	1.24
βA1-crystallin	9.23	6.19	−3.05
γB-crystallin	3.36	6.19	2.83
αB-crystallin	0.84	2.47	1.63
αA-crystallin	15.11	16.08	0.98
βB2-crystallin	0.84	8.66	7.82
βB3-crystallin	15.94	18.56	2.61
βA4-crystallin	3.36	2.47	−0.88
βB1-crystallin	36.92	54.43	17.51
βA2-crystallin	0.00	1.24	1.24
γA-crystallin	1.68	2.47	0.80
γC-crystallin	5.04	6.19	1.15
**1C: Normalized spectral counts for WI-UI Crystallins**			
βA1-crystallin	2.72	6.70	3.98
γB-crystallin	4.53	3.35	−1.18
αB-crystallin	0.91	0.00	−0.91
αA-crystallin	6.34	12.28	5.94
βB2-crystallin	2.72	6.70	3.98
γ F,E,D-crystallins	1.81	0.00	−1.81
βB3-crystallin	8.15	12.28	4.13
βA4-crystallin	0.00	1.12	1.12
βB1-crystallin	20.83	41.30	20.47
γC-crystallin	2.72	3.35	0.63

WS, Water-soluble; WI-US,Water insoluble-Urea soluble; WI-UI, Water insoluble-Urea soluble

**Table 2:** Mass spectrometric analyses for crystallins.

2A: Normalized spectral counts for WS Crystallins of M_r_ above 37 kDa			
	WT	βA3ΔG91	Difference (βA3ΔG91-WT)
γS-crystallin	116.46	142.83	26.37
βA1-crystallin	58.68	47.99	−10.68
γD-crystallin	217.81	252.52	34.71
γB-crystallin	210.70	226.24	15.54
γA-crystallin	71.12	78.84	7.72
αB-crystallin	408.06	429.63	21.57
αA-crystallin	575.20	625.02	49.82
βB2-crystallin	208.92	177.11	−31.81
γE-crystallin	225.81	285.66	59.85
γC-crystallin	179.58	236.53	56.94
βB3-crystallin	127.13	83.41	−43.72
βA4-crystallin	74.68	121.12	46.44
βB1-crystallin	36.45	41.13	4.68
βA2-crystallin	70.23	92.55	22.32
**2B: Normalized spectral counts for WI-US Crystallins of M**_**r**_ **above 37 kDa**			
γS-crystallin	25.92	36.32	10.41
βA1-crystallin	26.81	20.43	−6.38
γD-crystallin	71.49	71.51	0.02
γB-crystallin	84.90	77.19	−7.71
γA-crystallin	33.06	24.97	−8.09
αB-crystallin	103.66	141.89	38.23
αA-crystallin	186.77	154.37	−32.39
γE-crystallin	84.00	86.27	2.27
γC-crystallin	64.34	59.03	−5.32
βB3-crystallin	164.43	168	3.57
βA4-crystallin	63.45	60.16	−3.29
βB1-crystallin	58.09	73.78	15.70
βA2-crystallin	29.49	40.86	11.37
**2C: Normalized spectral counts for WI-UI Crystallins of M**_**r**_ **above 37 kDa**			
γS-crystallin	11.98	14.03	2.05
βA1-crystallin	14.97	14.04	−0.94
γD-crystallin	32.94	0.00	−32.94
γB-crystallin	54.89	37.07	−17.82
γA-crystallin	49.90	31.06	−18.84
αB-crystallin	78.85	160.31	81.46
αA-crystallin	136.73	241.47	104.73
	WT	βA3ΔG91	Difference (βA3ΔG91 – WT)
βB2-crystallin	39.92	37.07	−2.85
γE-crystallin	28.94	37.07	8.13
γC-crystallin	49.90	33.06	−16.84
βB3-crystallin	83.84	94.18	10.35
βA4-crystallin	24.95	25.05	0.10
βA2-crystallin	17.97	21.04	3.08
βB1-crystallin	39.92	53.10	13.18
**2D: Normalized spectral counts for WI-UI Crystallins of M**_**r**_ **below 20 kDa**			
γB-crystallin	17.43	8.35	−9.08
γA-crystallin	36.11	17.55	−18.56
αB-crystallin	4.98	21.72	16.74
αA-crystallin	118.29	126.96	7.87
βB2-crystallin	4.98	2.51	−2.47
γ F,E-crystallins	7.47	7.52	0.05
βB3-crystallin	2.49	25.90	23.41
βB1-crystallin	7.47	19.22	11.75
γS-crystallin	0.00	0.84	0.84

### Statistical analysis

Data are presented as mean ± SD. Experimental results from WT and βA3ΔG91 mice were analyzed for statistical significance using a two-tailed Student’s *t*-test. Significance was set at p < 0.05. Experiments were repeated at least twice to ensure reproducibility.

## Results

### Identification of Differentially Expressed Genes (DEGs) and molecular pathways affected in βA3ΔG91- vs WT- lenses

**Fig 1:**
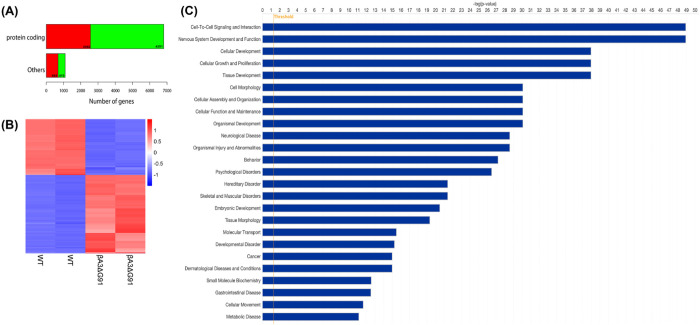
RNA sequencing analyses of βA3ΔG91 lenses relative to WT lenses. (A) Distribution profile of the lens transcripts detected in our RNA-Seq. analysis in βA3ΔG91 lenses compared to WT lenses. Genes were separated into protein-coding and non-protein-coding genes. Among the protein-coding genes, 2562 transcripts were upregulated (shown in red), and 4251 genes were downregulated (shown in green) in βA3ΔG91 lenses relative to WT lenses. In the non-coding genes, 683 genes were upregulated, and 415 were downregulated. (B) Heatmap of normalized log_2_FC values for DEGs in βA3ΔG91 lenses vs. WT lenses. Red color indicates genes with relatively high expression levels, and blue color shows relatively downregulated genes. (C) Ingenuity pathway analysis showing the top 25 biological pathways that are dysregulated in βA3ΔG91 mouse lenses compared to WT lenses.

In order to identify altered gene expression levels and explore dysregulated biological processes in the mouse lenses due to βA3ΔG91, we performed whole lens transcriptome analysis using mRNA-sequencing with Ingenuity Pathway and gene ontology analyses. Using a significance threshold of P < 0.05 and a log_2_ fold change (log_2_FC) of ≥ 1, we identified a total of 7911 DEGs between the βA3ΔG91- and WT- lenses. This set of DEGs comprised 3245 upregulated genes and 4666 downregulated genes in βA3ΔG91 lenses relative to the WT lenses. Further categorization revealed that the DEGs were classified into protein-coding genes and non-protein coding genes (Others), encompassing non-coding RNAs and pseudogenes. As shown in Fig 1A, among the protein-coding genes, 2562 transcripts were upregulated (shown in red), while 4251 genes were downregulated (shown in green) in βA3ΔG91 lenses compared to WT lenses. The non-coding genes comprised 683 and 415 up- and down-regulated transcripts, respectively (Fig 1A). All DEGs and the top 15 up- and down-regulated genes are listed as supplementary figures 1A 1B, and 1C, respectively (S1–S3 Figs). A heatmap of DEGs shows a distinct gene expression profile for the WT and βA3ΔG91 groups (Fig 1B). Additionally, Ingenuity Pathway Analysis was conducted to uncover affected biological functions in the βA3ΔG91 lenses relative to WT lenses. The top 25 affected biological processes are shown in Fig 1B, and they include “cellular development”, “cellular growth and proliferation” and “cellular movement” (Fig 1B). All DEGs of the specific biological functions mentioned above have been included as supplementary figures (S4–S6 Figs).

### Distribution of Differentially Expressed Genes (DEGs) in affected biological pathways

**Fig 2:**
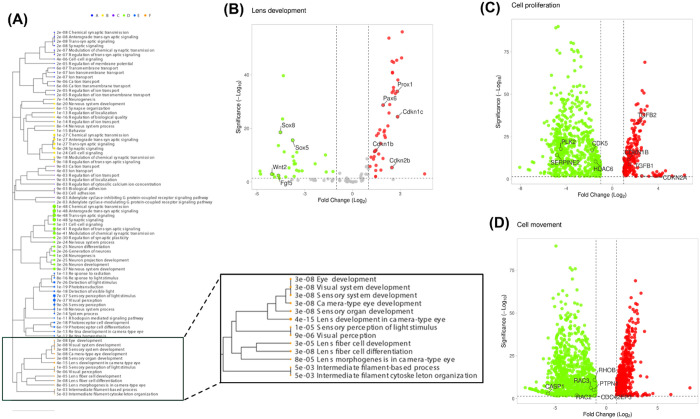
Distribution of Differentially Expressed Genes (DEGs) in affected biological pathways in βA3ΔG91 lenses relative to WT lenses. (A) Hierarchical clustering of RNA-seq profiles showing six distinct biological pathways affected in βA3ΔG91 lenses relative to WT lenses. Cluster F identifies defects in eye and lens development. (B to D) Volcano plots showing the distribution of genes that are involved in the following: (B) lens development, (C) cell proliferation, and (D) cell movement. Round circles represent genes. Green indicates downregulation, red indicates upregulation and grey represents non-statistically significant fold changes. A log_2_FC of 1 or more was considered significantly up- or down-regulated.

Hierarchical clustering from gene ontology analysis revealed six distinct pathway clusters, one of which was associated with lens development and lens fiber cell differentiation (Fig 2A). On examining the genes involved in lens development and differentiation, volcano plots showed downregulation of genes directly involved in lens development (Figs 2B) [8, 47–68]. These include *fgf*, *wnt* and *sox* genes and overexpression of *pax6, prox1* and *cdkn* genes.

We also examined the genes involved in cell proliferation (Fig 2C) and cellular migration (Fig 2D) by volcano plots. On examining specific genes responsible for cell proliferation, we found that inhibitory genes for epithelial cell proliferation such as *tgfβ* [69], c*dkn1b* [70], and *cdkn1c (p57*^*kip2*^) [62] were upregulated. Similarly, we noticed downregulation of *cdks*, *plk* and *hdac* genes which are known to induce epithelial cell proliferation. Probing specific genes for cell migration (Fig 2D) identified downregulation of *rho* and *rac* genes which drive the movement of cells during migration [54,71].

### Abnormal suture line pattern in βA3ΔG91 lenses

**Fig 3:**
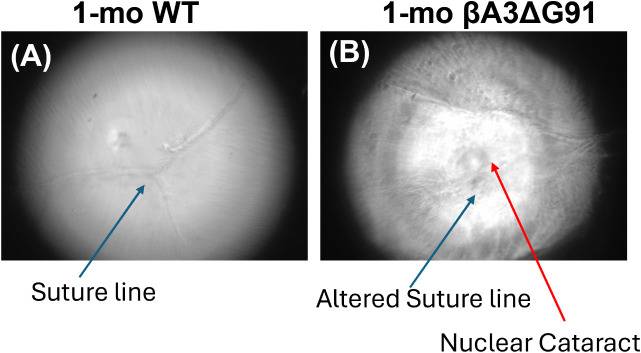
Suture line assessment in lenses of βA3ΔG91 vs. WT mice. (A) Suture line imaging revealed a normal “Y” pattern in 1-month-old WT lenses. (B) In 1-month-old βA3ΔG91 lenses, the suture line is disrupted.

The investigation of lens suture lines was prompted by our previous and current findings of reduced lens size, disorganization of lens fiber cells and dysregulated pathways in lens differentiation in βA3ΔG91 lenses relative to WT lenses [21]. During lens fiber cell differentiation, properly arranged lens fiber cells meet from opposite sides to form a “Y-shaped” suture line pattern, as seen in the 1-month-old WT lenses (Fig 3A). In contrast, βA3ΔG91 (Fig 3B) causes a noticeable shift in the Y-suture pattern towards the edge of the anterior lens, suggesting irregularities in lens fiber cell organization due to improper differentiation.

### Abnormal cell proliferation in βA3ΔG91 mice

**Fig 4:**
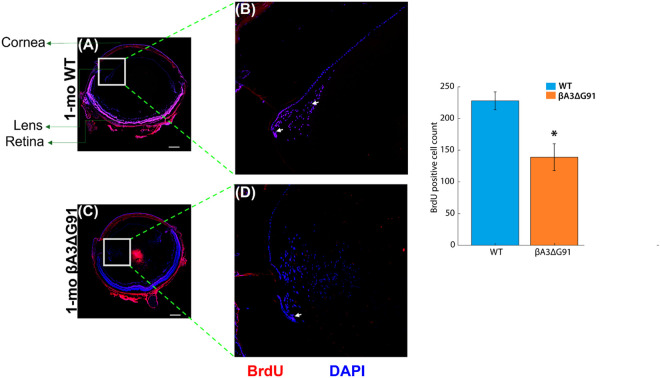
Analysis of cellular proliferation in βA3ΔG91 vs. WT mice lenses using BrdU assay. (A and B) BrdU labeling revealed greater staining in 1-month-old WT lenses. (C and D) βA3ΔG91 lenses, fewer cells were labeled by the BrdU dye. Scale bar: 200 µm. All images are shown in both 4X (A, C) and 20X (B, D) respectively. (E) Bar graph shows the quantification of BrdU-positive cells in 1-month-old βA3ΔG91 vs. WT lenses. *p < 0.05.

Abnormal suture line patterns and dysregulation of genes involved in cell proliferation by RNA-seq analyses of βA3ΔG91- compared to WT- lenses prompted further investigation into key processes critical for lens fiber cell differentiation. Lens epithelial cells (LECs) proliferate in the germinative zone and migrate toward the equator to undergo differentiation into fiber cells. To examine cell proliferation, we performed BrdU labeling on 1-month-old WT- and βA3ΔG91 mouse lenses. In WT lenses, most labeled cells were concentrated at the germinative and transition zones, shown by white arrows (Figs 4A and 4B), whereas similar regions of βA3ΔG91 lenses showed a reduction in the number of BrdU-labeled cells as indicated by white arrows (Figs 4C and 4D). The number of lens cells incorporating the BrdU dye was about 2X lower in βA3ΔG91 lenses relative to WT lenses (Fig 4E).

### Impaired LEC migration in βA3ΔG91 mice

**Fig 5:**
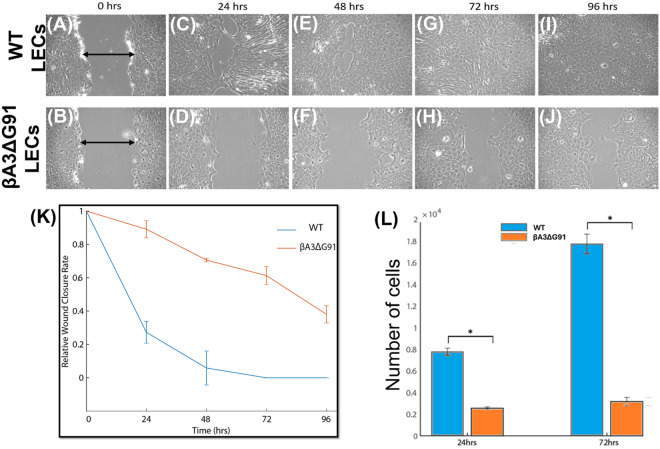
Cellular migration in LECs of βA3ΔG91 vs. WT mice using the scratch and transwell assays. (A to J) Scratch assay. (A and B) Representative images of scratches created in confluent LECs at time 0 in (A) WT and (B) βA3ΔG91 LECs. (C and D) After 24 hours, (C) WT cells significantly migrate towards the scratch area compared to (D) βA3ΔG91 LECs. (E and F) Images of LECs after 48 hours showing a faster rate of cell migration in the (E) WT LECs relative to (F) βA3ΔG91 LECs. (G and H) Images of LECs after 72 hours showing complete closure of the scratch gap in the (G) WT LECs compared to the (H) βA3ΔG91 LECs. (I and J) Complete gap width closure in (I) WT LECs compared to (J) βA3ΔG91 LECs after 96 hours following the scratch. (K) Line graph showing the normalized gap width over the different time points. (L) Transwell migration assay. The bar graph shows approximately a 3- and 6-fold increase in the number of migrated LECs in WT vs βA3ΔG91 at 24 and 72 hours respectively. *p < 0.05.

The above analyses were further confirmed via the scratch and transwell migration assays using LECs from WT and βA3ΔG91 mice. The scratch assay was done in three groups, each of WT and βA3ΔG91 confluent lens epithelial cells, denoted as 0 hr (Figs 5A and 5B). After 24 h (Figs 5C and 5D), 48 h (Figs 5E and 5F), 72 h (Figs 5G and 5H) and 96 h (Figs 5I and 5J) respectively, the cells were imaged to quantify the rate of migration. We observed that the migration of βA3ΔG91 LECs (Figs 5D, F, H, and J) was significantly reduced compared to WT LECs (Figs 5C, E, G, and I). By 48 h, the WT LECs had progressively migrated to cover the scratch (Fig 5E). The line-graph showing the normalized gap-width over the different time points (Fig 5K). A transwell migration assay was done to further validate the scratch assay (Fig 5L). Our results showed that there was a 3- and 6-fold increase in the number of migrated cells at 24 and 72 h respectively in WT LECs compared to βA3ΔG91 LECs (Fig 5L).

### Increased phalloidin staining in βA3ΔG91 lenses and LECs relative to WT

**Fig 6:**
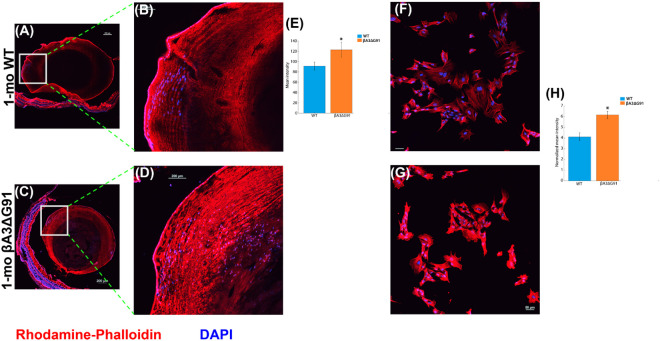
Elevated levels of F-actin in βA3ΔG91 lenses and LECs relative to WT. (A-D) Immunohistochemical analyses of F-actin using rhodamine-phalloidin in mouse lens sections. F-actin staining was relatively lower in the (A and B) WT lenses than in the (C and D) βA3ΔG91 lenses. Images are shown in 4X and 20X, respectively. (E) Bar graph showing quantification of F-actin staining intensity in βA3ΔG91 vs. WT lenses. (F and G) F-actin staining in the LECs, there is a decreased expression of F-actin in (F) WT mice relative to (G) βA3ΔG91 mice. (H) Bar graph showing quantification of F-actin staining intensity in βA3ΔG91 vs. WT LECs.

The actin cytoskeleton plays a crucial role in fiber cell organization, aside from their role in supporting cell proliferation and migration [72]. Specifically, cytoskeletal proteins support proliferation by providing a structural framework for cell division and regulating cell cycle progression. During cell migration, they facilitate cell movement by coordinating cellular dynamics and structural reorganization. The observation of decreased proliferation and migration prompted us to examine the expression of F-actin, an actin filament crucial for proper fiber cell organization in the lens. Rhodamine-Phalloidin was used to quantify F-actin in the WT (Figs 6A and 6B) and βA3ΔG91 lenses (Figs 6C and 6D) and LECs (Figs 6F and 6G, respectively). Rhodamine-Phalloidin staining showed that compared to WT lenses, which showed well-defined fiber cell strands with uniform staining (Fig 6A and 6B), βA3ΔG91 lenses revealed polymerized and abnormal F-actin distribution across the lens cortex (Fig 6C and 6D). Similarly, staining of lens epithelial cells (LECs) with Rhodamine-Phalloidin showed a well-defined meshwork and more organized F-actin filament strands in WT LECs (Fig 6F), which were not seen in βA3ΔG91 LECs (Fig 6G). These findings suggest that the βA3ΔG91 mutation may disrupt the cytoskeletal architecture in the lens, potentially contributing to altered fiber cell organization in βA3ΔG91 lenses, as observed from our previous studies [21].

### Molecular modeling and amyloidogenic properties of βA3-crystallin and βA3ΔG91

**Fig 7:**
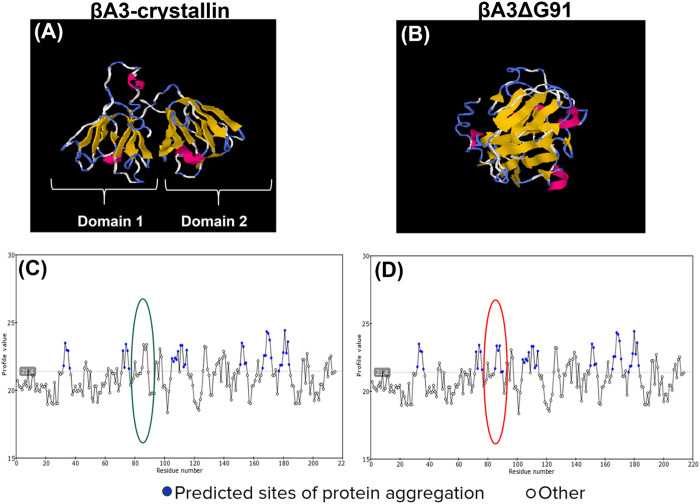
Protein modeling studies and prediction of amyloidogenic sites in βA3ΔG91 vs. native βA3-crystallin. (A and B) I-TASSER (Iterative Threading ASSEmbly Refinement) protein structure modeling predicted (A) an organized native βA3-crystallin structure compared to (B) a collapsed and misfolded βA3-crystallin structure due to G91-Deletion. (C and D) FoldAmyloidogenesis. Predicting regions of amyloidogenicity in (C) the native βA3-crystallin amino acid sequence vs. (D) βA3ΔG91 sequence reveals the emergence of several predicted sites of aggregation within residues between 80 to 100 where G91-deletion occurs.

Since Glycine-91 is located in a critical region of the βA3/A1-crystallin, known as the tyrosine corner [33] which is responsible for maintaining the overall fold stability of the protein, we determined the potential impact of G-91 deletion on the protein structure of βA3/A1-crystallin. Consistent with existing literature, the native βA3/A1-crystallin consists of four Greek key motifs that organize into two domains, connected by a short peptide (Fig 7A). Each Greek key motif comprises four sequentially arranged beta-strands, which were disorganized in the predicted βA3ΔG91 structure (Fig 7B). Using the I-TASSER protein structure prediction software, we demonstrated that G-91 deletion (ΔG91) could induce loss of structural integrity of the βA3-crystallin protein (Fig 7B). Destabilizing the βA3-crystallin protein fold may lead to aberrant aggregation and the formation of amyloid fibrils. To further explore this possibility, we performed a prediction analysis of the amyloidogenic regions in the native βA3/A1-crystallin and βA3ΔG91 sequences using the foldAmyloid software. From our results, unlike the native βA3-crystallin sequence (Fig 7C), the βA3ΔG91 sequence revealed the emergence of six additional amyloidogenic sites between amino acid residues 80 and 100, where the ΔG91 mutation occurs (Fig 7D). This suggested that βA3ΔG91 may cause aggregation, which could disrupt its function and cause cataract development, and this has been confirmed by mass spectrometry analysis from our previous studies showing that G-91 deletion causes insolubilization of βA3-crystallin [21].

### Amyloid staining using Thioflavin

**Fig 8:**
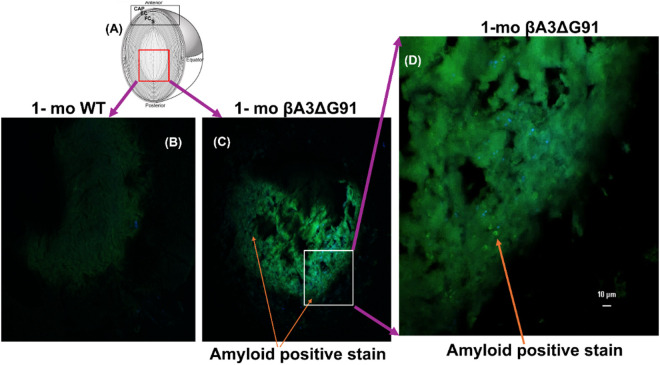
Amyloid staining using Thioflavin. (A) Schematic representation of a mouse lens and red box indicates the area where the images were taken from both WT- and βA3ΔG91 lenses. Thioflavin staining of the (B) WT lens and (C and D) βA3ΔG91 lens, respectively, shows a more pronounced signal in the (C and D) βA3ΔG91 lens, shown in 20x (Fig 8C) and 63x (Fig 8D) compared to the WT lens shown in 20X (Fig 8C).

To further validate the above findings, we performed Thioflavin S (ThS) staining to confirm the presence of amyloid fibrils in βA3ΔG91 lenses. Thioflavin S is a major histological stain used to detect any form of amyloid. These dyes bind to the characteristic β-pleated sheet conformation of amyloid. The representative regions of the mouse lens (1-month old) from both WT and βA3ΔG91 mice, as shown in Fig 8A, were analyzed using a confocal microscope after Thioflavin staining. Figs 8B–D show thioflavin staining of the WT lens (Fig 8B) and βA3ΔG91 lens (Figs 8C and 8D), respectively. Thioflavin staining was more pronounced in the βA3ΔG91 lens (Figs 8C and 8D) compared to the WT lens (Fig 8B). The orange arrow points to the green punctate staining in βA3ΔG91 (Figs 8C and 8D), which represents the amyloid staining in the βA3ΔG91 lenses. The molecular structural analysis highlights the aggregation and amyloidogenic properties of the βA3ΔG91 mutant protein. The increased green, fluorescent punctate staining in βA3ΔG91 lenses compared to the WT further confirms that mutation causes protein aggregation and contributes to cataract development in βA3ΔG91 mouse lenses.

### Size-exclusion HPLC analysis of lens crystallins

**Fig 9:**
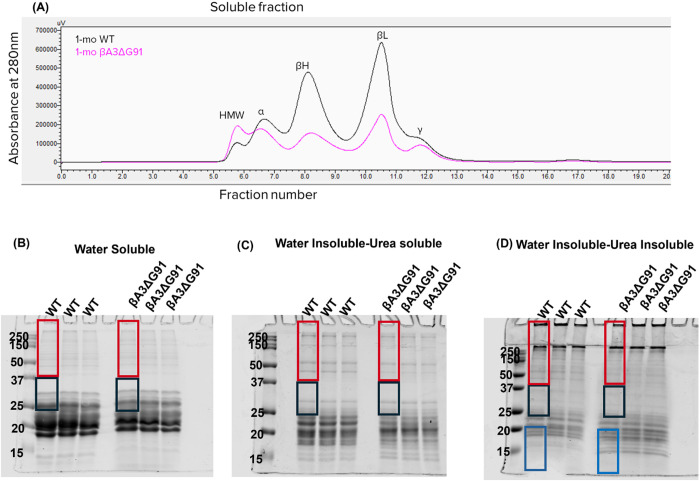
Size-exclusion HPLC and SDS-PAGE Analysis of βA3ΔG91 vs WT mouse lens proteins. (A) HPLC protein elution profiles at 280 nm of WS proteins from 1-month-old WT and βA3ΔG91 lenses show increased HMW proteins and reduced α-, β-, and γ-crystallin peaks in the βA3ΔG91 lenses relative to WT lenses. (B to D) Coomassie-blue stained gel of βA3ΔG91 vs WT lens proteins, (B) Water-soluble lens proteins (C) Water insoluble-urea soluble lens proteins and (D) Water insoluble-urea insoluble lens proteins in βA3ΔG91 vs WT lenses. Note that there were more degraded proteins (< 20 kDa) in the βA3ΔG91 water insoluble-urea insoluble fraction. Red, black and blue rectangles represent the areas that were analyzed for normalized spectral count quantification following mass spectrometry. All spectral count information are listed in Tables 1 and 2.

Based on our previous studies which showed that βA3ΔG91 changes from a soluble state to an insoluble state [21], we speculated that βA3/A1-crystallin insolubilization in βA3ΔG91 lenses might lead to aggregation of other crystallins due to aberrant crystallin-crystallin interactions [73,74]. To determine whether there are changes in the expression profiles of the crystallins in the lenses of WT and βA3ΔG91 mice. The water-soluble (WS) protein fractions of 1-month-old WT and βA3ΔG91 lenses were fractionated by a size-exclusion HPLC using a TSKgel G-4000PWxl column (Fig 9A). The comparative protein elution profiles at 280nm of βA3ΔG91 lenses showed an increased HMW peak and a decreased α-, βL-, βH-, and γ-crystallin peaks suggesting that the different crystallins were less abundant in their soluble forms and form HMW complexes and aggregates.

### Increased insolubilization and degradation of lens proteins in βA3ΔG91 lenses

We then employed mass spectrometric analyses to examine the protein levels in WT vs. βA3ΔG91 lenses (Fig 9). For this purpose, the lens proteins were identically fractionated into water-soluble (WS), water-insoluble-urea-soluble (WI-US), and water-insoluble-urea-insoluble (WI-UI) fractions as described in the Materials and Methods. Identical volumes of each fraction were analyzed using SDS-PAGE and stained with the Coomassie-blue dye. Examination of Coomassie blue-stained gels revealed a general decrease in band intensities across several molecular weights (Mr) in the WS fraction in the βA3ΔG91 lenses compared to the WT lenses. Notably, there was a marked reduction in band intensity around 25 kDa, which may indicate a loss of βA3-crystallin (Fig 9B). In the WI-US fraction, the βA3ΔG91 lenses showed an increase in the degraded protein fraction (below 20 kDa) compared to WT lenses (Fig 9C). Examination of the WI-UI gel revealed greater band intensities for proteins under 20 kDa in the βA3ΔG91 lenses. To quantify these changes, mass spectrometric analysis was performed. The gels were separated into three molecular weight fractions: (1) greater than 37 kDa, (2) 20–37 kDa, and (3) lower than 20 kDa.

Analysis of normalized spectral counts of crystallins between regions 20 to 37kDa revealed decreased levels of CRYAA, CRYBA1, and CRYBB1 in the WS fraction of the βA3ΔG91 lenses compared to WT lenses (Table 1A). In the WI-US fraction, the βA3ΔG91 group showed increased levels of most of the crystallins, including CRYBB1 and CRYBB2 (Table 1B). Also, in the WI-UI fraction, we observed increased levels of lens crystallins, such as CRYAA, CRYBB2, CRYBB1, and CRYBB3, in the βA3ΔG91 lenses compared to the WT (Table 1C). Together, these findings suggested a progressive insolubilization of CRYAA, CRYBB1, and CRYBB3 in βA3ΔG91 lenses compared to WT lenses.

To gain information on the solubility profiles of other crystallins, we focused on analyzing the aggregated crystallin region (>37 kDa). In the WS fraction, relative to WT lenses, βA3ΔG91 lenses showed increased normalized spectral counts for several γ-crystallins, as well as CRYAA, CRYAB, CRYBB1, and CRYBB3 relative to WT lenses (Table 2A). In the WI-US fraction, a subset of γ-crystallins (CRYGS and CRYGE), CRYAB, CRYBB1, and CRYBB3 showed increased levels in βA3ΔG91 lenses relative to WT lenses (Table 2B). Also, in the WI-UI fraction, all the crystallins mentioned above, along with CRYAA, showed increased levels, suggesting a shift towards insolubility in βA3ΔG91 lenses (Table 2C).

In the molecular weight (MW) region > 37 kDa, CRYAA, CRYAB, CRYBB1, CRYBB3, CRYGS, and CRYGE exhibited similar patterns of insolubilization. These crystallins are known to interact with CRYBA1, and aberrant interactions could lead to their aggregation.

Further analysis of degraded (<20 kDa) crystallins in the WI-UI fraction revealed elevated levels of fragments of CRYAA, CRYAB, CRYBB1, and CRYBB3 in βA3ΔG91 lenses compared to WT lenses (Table 2D), suggesting significant levels of crystallin degradation and loss of solubility. All data on spectral counts are listed as supplementary figures (S7–S20 Figs).

## Discussion

The precise pathogenic mechanisms underlying the development of congenital nuclear cataracts remain elusive. However, the underlying fact is that cataracts result from a series of molecular and biochemical events that undermine the structural stability of the lens fiber cells and their proteins. Our study elucidates the biochemical characteristics of βA3ΔG91 lenses, providing a foundation for understanding the pathogenesis of this condition. Our goal in this study was to (A) determine the affected genes and biological processes and pathways, (B) examine lens epithelial cell proliferation, migration, and differentiation, and (C) analyze changes in the solubility of lens crystallins in βA3ΔG91 lenses relative to WT lenses.

In rodent studies, βA3/A1-crystallin was first detected in the lens vesicle [75], suggesting its involvement in lens development. Findings from several studies suggest that βA3/A1-crystallin contributes to LFC differentiation, since its expression is induced during this process, and mutations in CRYβA1 significantly disrupt differentiation-related events, including migration [76,77]. Using RNA-sequencing and Ingenuity Pathway Analysis, we conducted comprehensive gene expression analyses of WT vs. βA3ΔG91 mice lenses. Consistent with other findings, Ingenuity Pathway Analysis in βA3ΔG91 vs. WT lenses highlighted key dysregulated pathways for lens development (Fig 2B), cell proliferation (Fig 2C), and cell movement (Fig 2D). We observed altered expression of key genes involved in lens development and fiber cell differentiation, including *pax6*, *prox1*, *sox, fgf, wnt, tgfβ, cdk and cdkn* genes [50–52,56,62–65,68,78–80]. *Pax6* upregulation has been linked to microphthalmia, which aligns with our findings in βA3ΔG91 mice [56]. *Prox1* promotes cell cycle exit in mouse retina progenitor cells [60]. *Sox, fgf,* and *wnt* genes are expressed during lens embryological development, whereas *cdkn* acts as a tumor suppressor to inhibit proliferation. Cell proliferation and migration are important events that precede lens fiber cell differentiation. The fewer BrdU-positive cells in βA3ΔG91 lenses (Fig 4) likely reflect the upregulation of genes inhibiting mitosis. This decrease in mitotic activity can impair the regular progression of lens epithelial cells toward the equatorial region, where they are supposed to differentiate into new fiber cells. βA3/A1-crystallin is highly expressed in differentiated RPE cells but not in undifferentiated ones [18], suggesting its involvement in maintaining epithelial characteristics. CRYβA1-knockout lenses lose their epithelial cell identity, affecting key processes such as proliferation and migration [18–20].

Lens fiber cell (LFC) differentiation involves the proliferation and migration of lens epithelial cells (LECs) toward the equatorial region, where these LECs transform into highly specialized LFCs. During this process, the LFCs undergo significant changes, including loss of cytoplasmic organelles via autophagy, and structural and cytoskeletal reorganization. As a key regulator of mTORC1, βA3/A1-crystallin plays a critical role in maintaining cellular homeostasis under stress and has been implicated in regulating oxidative stress, lysosomal function/autophagy, apoptosis, and inflammation [81]. Previous studies have shown that the absence of βA3/A1-crystallin in RPE cells activates mTORC1 signaling and inhibits autophagy, while its overexpression reverses these effects [19,82]. We demonstrated attenuation of nuclei degradation and autophagy disruption in βA3ΔG91 lenses relative to WT lenses from our previous studies [21]. Given the established role of βA3/A1-crystallin as a biological modulator of mTORC1, it is possible that βA3ΔG91 would affect other biological processes regulated by mTORC1 signaling, including cell proliferation, migration, and maintaining cytoskeletal architecture.

From our previous findings, autophagy dysregulation in βA3ΔG91 mouse lenses causes an accumulation of damaged organelles and aggregated proteins in the lens cells, which would cause aberrant properties of the lens epithelial and fiber cells. Also, autophagy helps in the turnover of cytoskeletal components [83] needed for migration and fiber cell organization. We observed decreased proliferation and migration of LECs (Figs 4 and 5). Phalloidin staining also suggests abnormal polymerization of F-actin in βA3ΔG91 compared to the normal filamentous structure seen in the WT lenses (Fig 4). Studies on CRYβA1-mutant- Nuc1 rat lenses have shown poor migration of lens cells and retention of nuclei in mature lens fibers [76]. This destroys the integrity of the lens and the organization of the fiber cells. Moreover, disruption of the cytoskeleton can activate apoptosis, as shown in our previous study [21]. Apoptosis destroys the lens cells, creating inconsistencies in the fiber cell architecture, which can lead to the formation of abnormal suture lines in βA3ΔG91 lenses relative WT lenses as illustrated in Fig 3, and subsequent cataract development. The lens suture lines are seam-like structures formed by the overlapping tips of elongated fiber cells from opposite directions [84]. These patterns reflect the precise elongation and migration of fiber cells within the lens. Any disruptions in this process, such as defects in fiber cell elongation or migration, can result in irregular or excessive suture branches [85]. Such abnormalities in suture formation are thought to impact lens optical quality and have been associated with cataract development [86,87].

The crystalline lens is the most protein-dense tissue in the body, creating a susceptible environment for protein aggregation even though the lens crystallins possess unique characteristics that make them extremely stable, soluble, and long-lived [88]. βA3ΔG91 disrupts the proper folding of βA3/A1-crystallin, as shown in Fig 7, compromising its stability and solubility [33] and potentially impairing crystallin-crystallin interactions, leading to protein aggregation [89,90]. Glycine-91 occupies a highly conserved site in the βA3-crystallin sequence called the tyrosine corner that stabilizes the native protein fold [91]. A previous study has shown that the substitution of a conserved glycine in a protein sequence can facilitate protein aggregation [92]. We also showed that βA3ΔG91 affected the solubility of other crystallins (Tables 1 and 2), particularly αA-, αB-, βB1-, and βB3-crystallins, considering their known interactions with one another. A mutation in one β-crystallin gene can alter the overall profile of assembled β-crystallin oligomers in the lens [33]. Also, acidic crystallins are solubilized and stabilized by their interactions with basic β-crystallins, and disruptions in these interactions have been linked to protein aggregation and cataract development [93]. Our previous findings from βA3ΔG91 mice show that the mice have a congenital nuclear cataract [21].

Overall, our study shows the molecular changes associated with cataract development in βA3ΔG91 mice. Our findings suggest that βA3ΔG91 disrupts lens fiber cell differentiation and leads to the insolubilization of lens crystallins, potentially leading to cataract formation. This study highlights the importance of protein solubility and normal lens differentiation in maintaining lens homeostasis and provides insights into the pathogenesis of cataracts associated with G-91 deletion in βA3/A1-crystallin.

## Supporting information

S1 Fig RNA-seq data showing Differentially Expressed Genes (DEGs). (XLSX)

S2 Fig RNA-seq data showing top 15 upregulated genes. (XLSX)

S3 Fig RNA-seq data showing top 15 downregulated genes. (XLSX)

S4 Fig RNA-seq data showing the distribution of lens development genes. (XLSX)

S5 Fig RNA-seq data showing the distribution of cell proliferation genes. (XLSX)

S6 Fig RNA-seq data showing the distribution of cell movement genes. (XLSX)

S7 Fig Mass Spectrometry data showing wildtype water soluble spectral counts between M_r_ 20–37 kDa. (CSV)

S8 Fig Mass Spectrometry data showing βA3ΔG91 water soluble spectral counts between M_r_ 20–37 kDa. (CSV)

S9 Fig Mass Spectrometry data showing wildtype water insoluble-urea soluble spectral counts between M_r_ 20–37 kDa. (CSV)

S10 Fig Mass Spectrometry data showing βA3ΔG91 water insoluble-urea soluble spectral counts between M_r_ 20–37 kDa. (CSV)

S11 Fig Mass Spectrometry data showing wildtype water insoluble-urea insoluble spectral counts between M_r_ 20–37 kDa. (CSV)

S12 Fig Mass Spectrometry data showing βA3ΔG91 water insoluble-urea insoluble spectral counts between M_r_ 20–37 kDa. (CSV)

S13 Fig Mass Spectrometry data showing wildtype water soluble spectral counts of M_r_ > 37 kDa. (CSV)

S14 Fig Mass Spectrometry data showing βA3ΔG91 water soluble spectral counts of M_r_ > 37 kDa. (CSV)

S15 Fig Mass Spectrometry data showing wildtype water insoluble-urea soluble spectral counts of M_r_ > 37 kDa. (CSV)

S16 Fig Mass Spectrometry data showing βA3ΔG91 water insoluble-urea soluble spectral counts of M_r_ > 37 kDa. (CSV)

S17 Fig Mass Spectrometry data showing wildtype water insoluble-urea insoluble spectral counts of M_r_ > 37 kDa. (CSV)

S18 Fig Mass Spectrometry data showing βA3ΔG91 water insoluble-urea insoluble spectral counts of M_r_ > 37 kDa. (CSV)

S19 Fig Mass Spectrometry data showing wildtype water insoluble- urea insoluble spectral counts of M_r _< 20 kDa. (CSV)

S20 Fig Mass Spectrometry data showing βA3ΔG91 water insoluble-urea insoluble spectral counts M_r _< 20 kDa. (CSV)

S21 raw files Original uncropped images of Coomassie stained water soluble, water insoluble-urea soluble and water insoluble-urea insoluble gels used for Mass Spectrometry analyses. (PDF)
