# Supplementary figures and images for: G91-deletion in βA3/A1-crystallin induces cellular and molecular changes in mouse lenses leading to congenital cataract development
Source: PLoS One. 2025 Jul 7;20(7):e0326305. doi: 10.1371/journal.pone.0326305 (PMC12233310; doi:10.1371/journal.pone.0326305)

Water Soluble

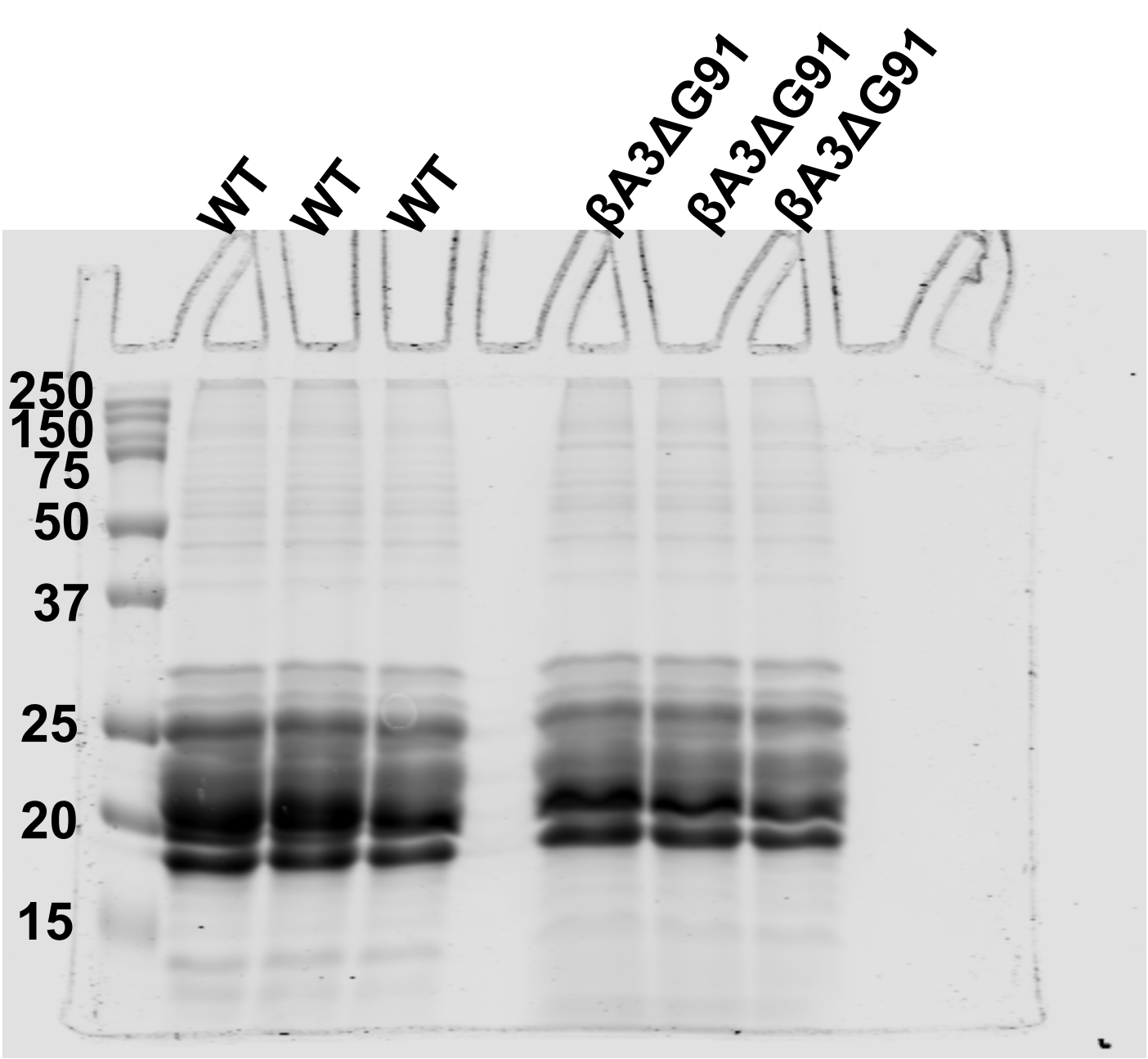

Water Insoluble-Urea Soluble

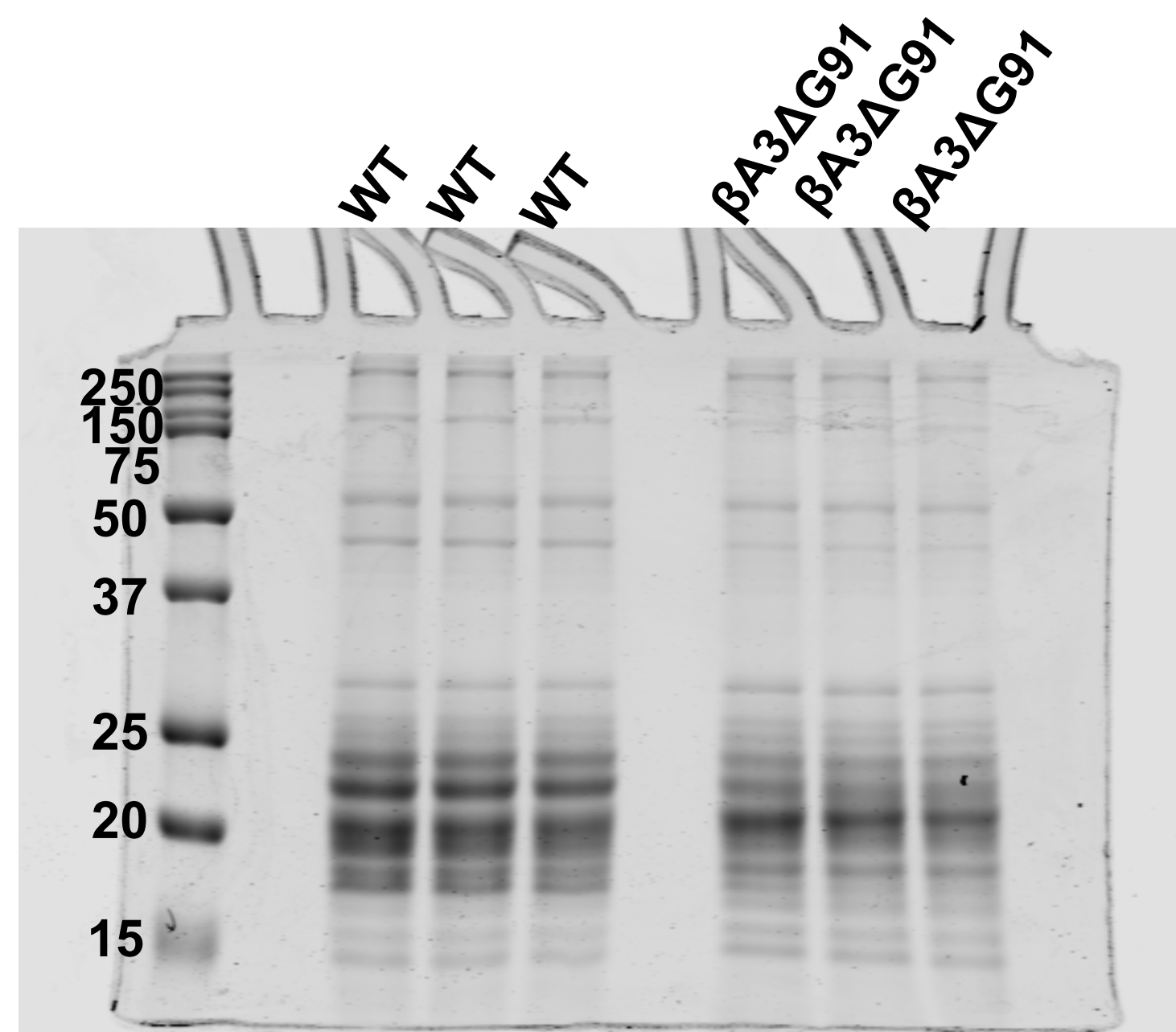

Water Insoluble-Urea Insoluble

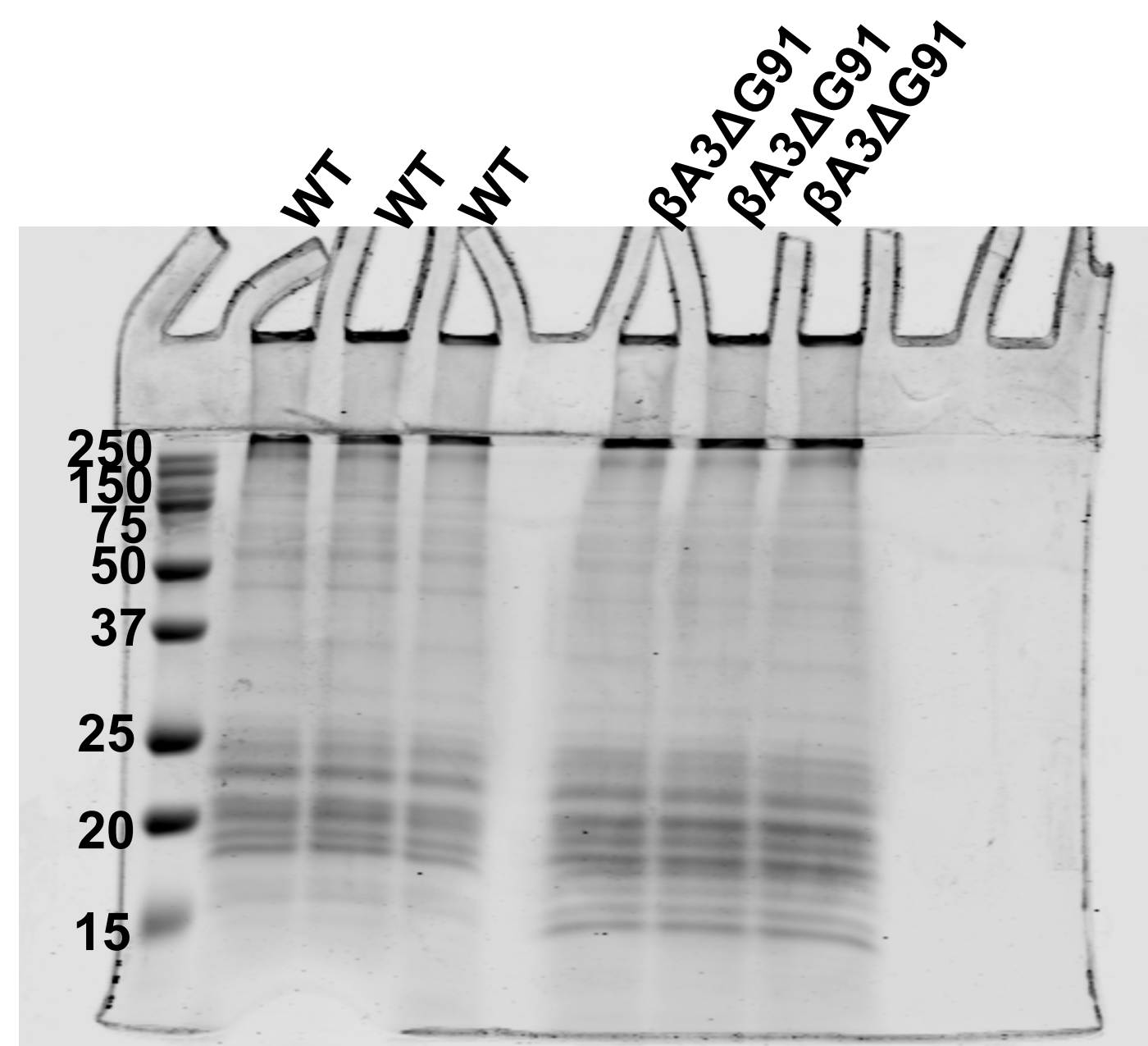

Supplement: S21 raw files — (PDF) [file pone.0326305.s021.pdf]
